# PM_2.5_ air pollution inequities in the US by sector and state: Past trajectories and future directions

**DOI:** 10.1126/sciadv.aed7425

**Published:** 2026-06-10

**Authors:** Bujin Bekbulat, Arushi Sharma, Joshua S. Apte, Robert Bullard, Libby H. Koolik, Esther Min, Rachel Morello-Frosch, Manuel Pastor, Regan Patterson, Allen L. Robinson, Manuel Salgado, Alper Unal, Nicolas Wedekind, Julian D. Marshall

**Affiliations:** ^1^ Department of Civil and Environmental Engineering, University of Washington, Seattle, WA 98195, USA.; ^2^ Department of Civil and Environmental Engineering, University of California, Berkeley, Berkeley, CA 94720, USA.; ^3^ Barbara Jordan-Mickey Leland School of Public Affairs, Texas Southern University, Houston, TX 77004, USA.; ^4^ Front & Centered, Seattle, WA 98121, USA.; ^5^ Department of Environmental and Occupational Health Sciences, University of Washington, Seattle, WA 98195, USA.; ^6^ School of Public Health and Department of Environmental Science, Policy and Management, University of California, Berkeley, Berkeley, CA 94720, USA.; ^7^ Equity Research Institute, University of Southern California, Los Angeles, CA 90015, USA.; ^8^ Department of Civil and Environmental Engineering, University of California, Los Angeles, Los Angeles, CA 90095, USA.; ^9^ Department of Mechanical Engineering, Colorado State University, Fort Collins, CO 80523, USA.; ^10^ WE ACT, Washington, DC 20001, USA.; ^11^ Eurasia Institute of Earth Sciences, Istanbul Technical University, Istanbul 34467, Türkiye.; ^12^ Earthjustice, Seattle, WA 98104, USA.

## Abstract

On average in the United States (US), low-income communities and communities of color face disproportionate burdens of air pollution and associated health impacts. Much of the effort to reduce these disparities will need to occur at the state level, through the targeting of specific emission sources and exposures. We present the first analysis of exposure disparities in the US that systematically investigates temporal trends, disaggregated by economic sector and state. Our approach combines a recently released time-series (2002 to 2019) emission inventory (EQUATES) with a computationally efficient air pollution model (InMAP Source-Receptor Matrix) to predict annual average exposure to ambient PM_2.5_ in each census tract in the contiguous US, disaggregated by emission sector. We find that, in general, absolute disparities have decreased, but relative disparities remain largely unchanged. This pattern holds for some sectors (e.g., transportation and electricity) but not others (e.g., disparities are largely unchanged for agriculture emissions and are generally increasing for residential and cooking emissions). Some state-sector combinations exhibit distinct patterns (e.g., most-recent disparities in New York are dominated by cooking and in Florida by construction). Of all sectors, industrial emissions contribute the largest exposures and the greatest exposure disparities. Agriculture and residential energy use are also large contributors to most-recent disparities, underscoring that inequities persist in sectors historically subject to less regulatory focus. Overall, our findings reveal how disparities have changed during 2002 to 2019 for each sector-state combination and elucidate which sectors each state should focus on to address recent disparities.

## Teaser

Air pollution disparities across US states and sectors varied from 2002 to 2019, declining in some areas but persisting elsewhere.

## INTRODUCTION

Levels of ambient fine particulate matter (PM_2.5_) in the US have declined substantially since the 1970 Clean Air Act (*1*). Nevertheless, PM_2.5_ exposure still remains associated with ~65,000 premature deaths annually (*2*). Health effects occur even below current regulatory standards, with no evidence of a safe threshold (*3*–*6*). These health burdens have long been unevenly distributed: Communities of color and socioeconomically disadvantaged groups consistently experience higher-than-average PM_2.5_ exposures (*7*–*13*). Over time, levels of air pollution have generally declined nationwide for all groups. However, the relative ranking of who is most exposed has remained largely unchanged, with the same groups bearing the greatest burden (*14*).

Regulatory actions for air pollution typically address emissions by sector and jurisdiction. Recent environmental justice (EJ) initiatives and state laws aim to reduce inequities in exposure and health (*15*, *16*). The state level is where much of the policy has varied and likely will continue to vary, especially in light of the current federal retrenchment away from EJ policies. However, policymakers lack systematic evidence on which sectors drive disparities within each state and how those contributions have changed over time. It is also unclear whether progress for a specific sector has translated into more equitable outcomes and how these patterns vary across individual states. This evidence is critical for designing interventions that simultaneously reduce overall pollution and advance EJ.

Previous studies have examined PM_2.5_ exposure disparities using diverse data sources and models, but most are limited to a single time point, sector, or geographic scale (*12*, *13*, *17*–*21*). National analyses often assess all sectors for a single year (*17*, *18*), while temporal studies typically focus on one sector (*19*), and multisector, multiyear analyses are frequently confined to individual states or cities (*20*). One study examined long-term changes in emissions and county-level disparities across sectors (*21*) but did not assess population exposure. Recent studies project how hypothetical sectoral emission reductions could influence disparities (*22*–*25*). Existing work does not reveal how the sources driving exposure disparities have evolved jointly across sectors, states, and time.

This paper addresses that gap. We present the first comprehensive state-level temporal analysis of modeled disparities in exposure by race-ethnicity and income, disaggregated across 11 major economic sectors. We quantify absolute disparities (units: micrograms per cubic meter) and relative disparities (units: %) in annual-average exposure, defined relative to the overall population average within each state, and track their evolution over nearly two decades. This allows us to identify which sectors have seen reductions or increases in disparities and where progress has stalled. We focus on ambient, annual-average anthropogenic PM_2.5_ during 2002 to 2019, based on an available time-series emission inventory. An important advancement is disaggregating time trends by economic sector and US state. This advancement is made possible by two developments. First, highly efficient air pollution models make large-scale analyses computationally feasible. Second, a recently developed emission inventory applies consistent calculations across time and space, allowing observed changes to represent real trends rather than methodological artifacts. By linking exposure disparities to sectoral emissions at the state level, our analysis identifies important drivers of exposure inequities and offers actionable evidence to guide future air quality and EJ policies.

## RESULTS

Our analysis reveals some patterns that are consistent with prior literature and some that have not been previously documented. As described below, results reflect exposures in each state attributable to emissions anywhere. We highlight here six specific findings.

**Fig. 1.**
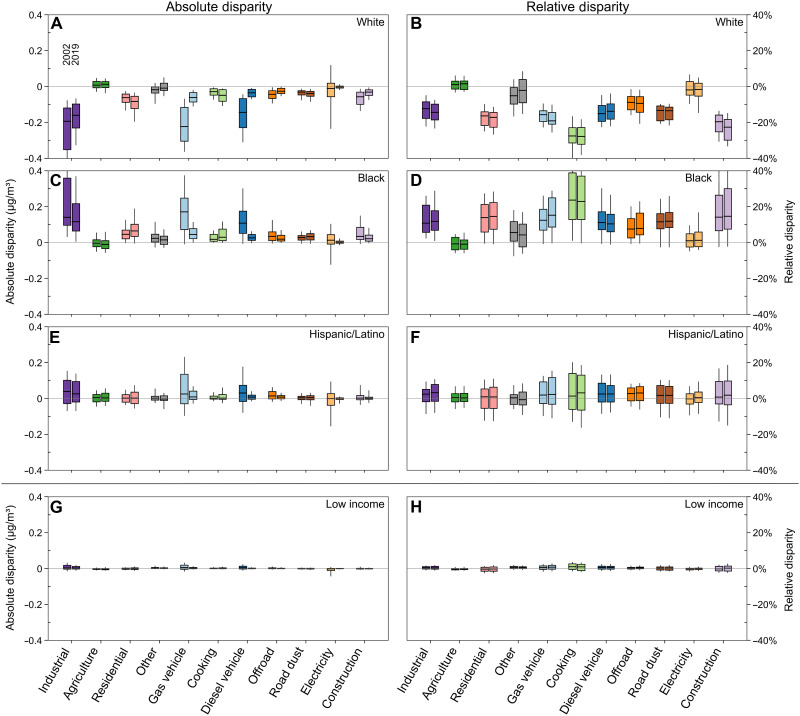
Changes in absolute and relative disparities in PM_2.5_ exposure by sector, race-ethnicity, and income. Box plots of absolute (left column) (**A**, **C**, **E**, and **G**) and relative (right column) (**B**, **D**, **F**, and **H**) within-state PM_2.5_ exposure disparities, for each of the 11 economic sectors. Each box plot is in pairs: 2002 (left box in each pair) and 2019 (right box). The top three rows show disparities by race-ethnicity (white, Black, Hispanic/Latino), i.e., the state-level difference in population-weighted average exposure between that group and the overall population; the bottom row shows disparities by economic group (low income). Each box summarizes the distribution across states (median state; among states: interquartile range and 10th/90th percentiles). Figure S1 displays results for additional racial-ethnic and income groups. Disparities are larger by race-ethnicity than by income. Differences between the paired box plots (i.e., 2002 versus 2019) indicate historical trajectories; remaining disparities (in 2019) indicate future targets.

First, across economic sectors from 2002 to 2019, modeled state-level exposure disparities were consistently and substantially larger by race-ethnicity than by income (Fig. 1; additional groups in fig. S1).

Second, in general, absolute disparities narrowed over time, yet relative disparities changed little—indicating that while overall exposure gaps narrowed, proportional gaps persisted (Fig. 1). Figure 1 displays paired box plots for 2002 and 2019, such that temporal patterns can be assessed by comparing the second box (2019) to the first box (2002) within each pair. For absolute disparities, the second box tends to be lower than the first; for relative disparities, this decline is not observed. For example, for industrial emissions in the median state (Fig. 1), the absolute disparity in population-weighted average mean exposures for the Black population versus for the overall population declined from 0.14 μg/m^3^ (2002) to 0.11 μg/m^3^ (2019), while relative disparities were approximately unchanged: ~12% (2002 and 2019; see table S1). In comparison, those same disparities for low-income populations were more than an order of magnitude smaller and nearly unchanged over time (absolute: ~0.004 μg/m^3^; relative: ~0.5%; see Fig. 1 and table S1).

**Fig. 2.**
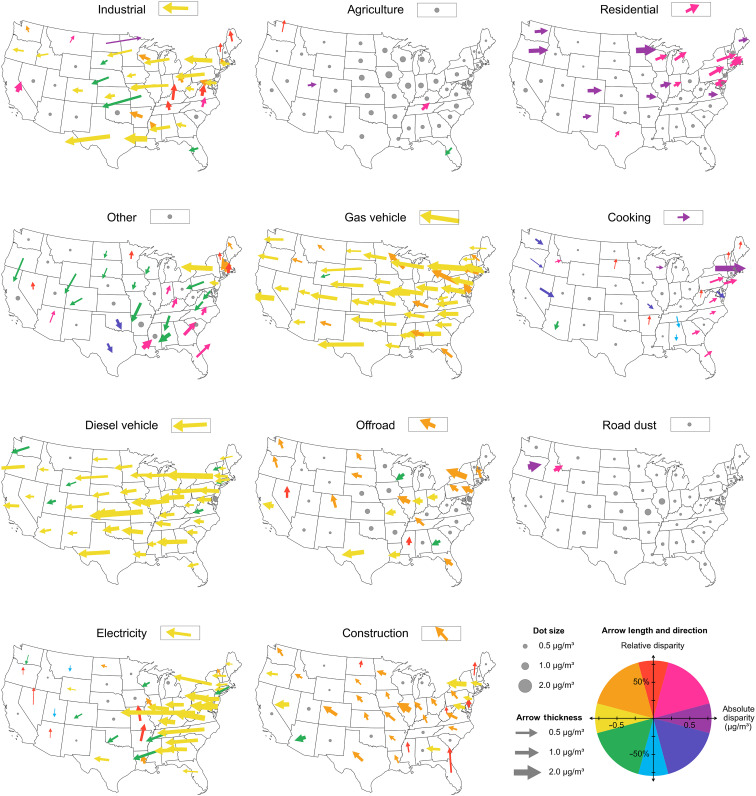
Changes in absolute and relative disparities in PM_2.5_ exposure by state and economic sector. For each state and economic sector, the arrow indicates the historical change, 2002 to 2019, in absolute (*x*-axis direction) and relative (*y*-axis direction) PM_2.5_ exposure disparity by race-ethnicity. Disparities shown are the state-level difference in population-weighted average exposure between the most-exposed group and the overall population; for example, if the arrow points to the top right [pink color; e.g., residential emissions in New York (NY)], relative and absolute disparity are increasing; if the arrow points horizontally to the left (yellow color; e.g., diesel vehicles in NY), absolute disparity is decreasing while relative disparity is unchanged. Arrow length indicates the amount of change in disparity (arrow height: relative disparity; width: absolute disparity). If relative and absolute disparity are nearly unchanged (e.g., agricultural emissions in NY), the icon is a gray dot. Arrow thickness (or dot size) reflects the sector’s population-weighted average PM_2.5_ exposure in 2010. At the top of each map (small box next to map heading), an arrow or dot displays the average among states. Figure S2 provides analogous results by income. Disparities are larger by race-ethnicity than by income.

Third, the reduction in absolute but not relative disparities (i.e., finding #2 above) holds for some sector-state combinations [e.g., gasoline and diesel vehicles in most states, electricity in many Eastern states, and industrial sources in a few states (e.g., New York, Texas, and Indiana)] but not others (Fig. 2). This pattern is seen in Fig. 2 by the yellow arrows pointing left. However, for residential and cooking emissions in several states (e.g., New York, Oregon, and Minnesota) and for industrial emissions in North Dakota, arrows in Fig. 2 are pink and purple, pointing right, indicating that absolute disparities have increased. In nearly all states, it (i.e., finding #2) also does not hold for agriculture and road dust, for which disparities have changed little over time, as signified in Fig. 2 by gray dots. For construction-related emissions in Florida and industrial emissions in Kentucky, for example, absolute disparities increased, and relative disparities were relatively unchanged; this alternative pattern is indicated by red upward arrows in Fig. 2.

**Fig. 3.**
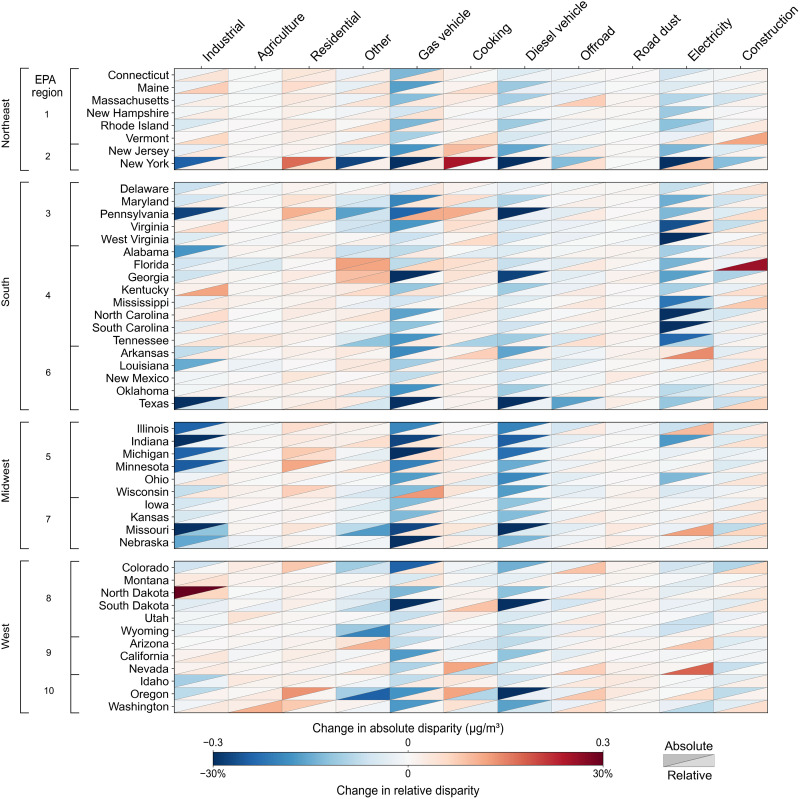
Heat maps of changes in absolute and relative disparity in PM_2.5_ exposure, from 2002 to 2019, by state and economic sector. Disparities are defined as in Fig. 2. Cell colors show changes from 2002 to 2019, in absolute (top-left triangle of each cell) and relative (bottom-right of each cell) disparity by race-ethnicity. Figure S3 provides analogous results by income. Disparities are larger by race-ethnicity than by income.

Heatmaps [Fig. 3 (race) and fig. S3 (income)], wherein each cell represents a state-sector combination, provide a complementary view, revealing differences across states and US Environmental Protection Agency (EPA) regions. Consistent with the different trends for absolute versus relative disparities stated above (i.e., finding #2), in Fig. 3, many cells have darker top triangles (i.e., more change for absolute disparities) and lighter bottom triangles (i.e., less change for relative disparities). Large reductions (Fig. 3, dark-blue color) are more common for absolute disparities than for relative disparities.

Fourth, select state-sector combinations exhibit large increases in absolute and relative disparities. This outcome would be seen in Fig. 3 as a cell (top and bottom triangles) that is dark red, such as cooking in New York and industrial sources in North Dakota. This outcome is rare but important.

**Fig. 4.**
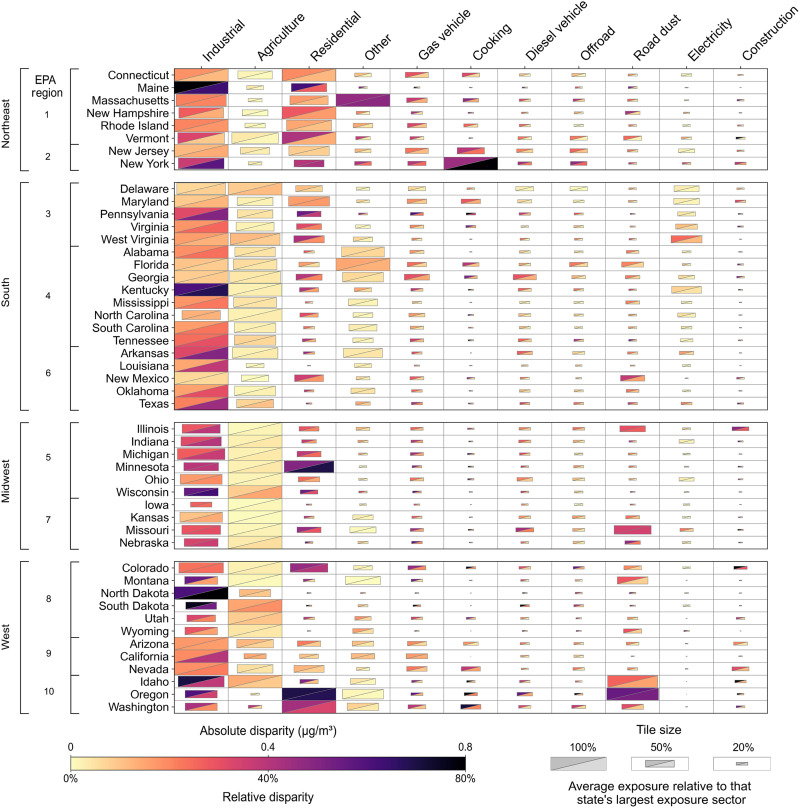
State and sector heat maps of remaining PM_2.5_ exposure disparities in 2019. Disparities are defined as in Figs. 2 and 3. Like in Fig. 3, top left of each cell displays absolute disparity by race-ethnicity, bottom right of each cell displays relative disparity by race-ethnicity. Here (unlike in Fig. 3), icon size is proportional to the population-average exposure for that economic sector and state, thereby visually emphasizing the sectors that are more important to the current (year-2019) exposure disparities in that state. Triangle size is scaled within each state, relative to the largest sectoral population-average exposure in that state. For example, if two tiles for a state have a size of 100%, that indicates that they are “tied for largest sector” within that state; a tile size of 50% indicates that the exposures for that sector are 50% of the exposures for the largest sector in that state. This figure shows future targets, i.e., changes that would be needed to eliminate existing (in year-2019) disparities in each state. Figure S4 provides analogous results by income. Disparities are larger by race-ethnicity than by income.

Fifth, a future-looking perspective highlights where disparities remain in 2019 (Fig. 4). Opportunities exist for meaningful mitigation for each state-sector combination [Fig. 4 (race) and fig. S4 (income)]. In this future-looking view, industrial emissions are the largest source of remaining disparities in nearly all states. Industrial emissions combine the largest modeled exposures with the greatest disparity reductions still required for equity. Emissions from agriculture and residential energy use follow as additional major contributors to remaining disparities, underscoring that inequities persist even in sectors historically subject to less regulatory focus.

Sixth, several specific state-sector combinations are outliers and provide opportunities for future improvement (see Figs. 2 to 4). For example, in New York, cooking is the largest contributor to absolute and relative disparities and also represents the state’s highest population-weighted exposure. In a handful of states (e.g., Washington, Oregon, Minnesota, New Hampshire, and Vermont), residential emissions are a large contributor to year-2019 disparities, likely reflecting the prevalence of residential wood smoke. These patterns underscore that while overall PM_2.5_ exposures have fallen during 2002 to 2019, achieving environmental equity will require continued effort, with a focus on industrial, agricultural, and residential emissions nationwide, and targeted interventions for persistent local outliers such as cooking emissions in New York.

## DISCUSSION

This analysis provides the first state- and sector-specific assessment of PM_2.5_ exposure disparities by race-ethnicity and income across nearly two decades. The findings are both diagnostic and actionable: They identify which sectors and states have contributed most to disparities, how these contributions have changed over time, and where disparities remain. Results here identify state-sector combinations that do and do not follow the dominant pattern (absolute disparities decrease, relative disparities unchanged) reported in the literature (i.e., finding #2 above). This dominant pattern has previously been reported for total concentrations (*7*–*11*); our results provide attribution (i.e., are disaggregated by source), revealing that the dominant pattern holds for only some state-sector combinations. The dominant pattern is consistent with emissions decreasing but structural inequities remaining largely unchanged.

Under the established concentration-response relationships for PM_2.5_, absolute differences in attributable health risks are related to absolute (rather than relative) differences in exposure (*26*–*28*). The substantial declines in absolute exposures and exposure disparities observed here therefore imply meaningful reductions in PM_2.5_-attributable health risk (and absolute risk disparity) over time, consistent with the broader improvements in air quality achieved in the United States (*29*, *30*). Air-quality gains have benefitted all groups, even as the persistence of relative disparities indicates that marginalized populations continue to experience disproportionately higher exposure relative to population averages (*31*–*34*). This suggests that additional health benefits, including reducing inequities in air pollution-related health effects, could be achieved by further reducing remaining inequities in exposure.

Some findings here may be unexpected. Industrial emissions were previously (i.e., in 2002) not the leading contributor to disparities, but recently (in 2019) they are the largest contributor (Fig. 4) in most states, rivaling or exceeding on-highway vehicles. Residential energy use (including residential wood smoke) and agriculture also remain major contributors (*17*, *18*, *35*–*40*). These results suggest that reducing inequities in the future will require a broader regulatory scope, extending beyond historically prioritized sources such as power plants, industry, and vehicles (*41*, *42*), to include sectors such as agriculture (*43*, *44*), and residential combustion (*45*, *46*). Some high-impact state-sector results are atypical [e.g., cooking (New York) and construction (Florida)], highlighting the importance of tailoring actions to state-specific conditions.

Results here provide an exposure-focused investigation of disparities, i.e., the disparities in each state attributable to national emissions from each sector. Future work could investigate alternative framings such as emissions-focused (i.e., national disparities attributable to sectoral emissions in each state) or local-emissions-focused (i.e., disparities in each state attributable to same-state sectoral emissions).

As with any analysis of this scope, uncertainties remain. Estimated disparities vary across space and time and depend on emission inventories that are uncertain and subject to reporting limitations and methodological choices. Additional uncertainty arises from air quality modeling assumptions in Intervention Model for Air Pollution (InMAP)/InMAP Source-Receptor Matrix (ISRM), including potential spatial variability in model performance and differences in uncertainty across source sectors and regions. These assumptions and uncertainties are documented elsewhere (*11*, *47*–*49*). A strength of this study is the use of the EPA’s Air Quality Time Series Project (EQUATES) emission inventory, which applies a consistent methodology across states, sectors, and years, enabling systematic comparison even where absolute estimates are uncertain. EQUATES was specifically developed by EPA using consistent methods across time and locations. Similarly, we apply the same spatial allocation methods and surrogates consistently across time and locations. Therefore, observed spatial or temporal patterns are likely not “artifacts,” i.e., are not merely a reflection of methodological inconsistencies. Nevertheless, recognizing the potential for unknown uncertainties, state-sector outliers (e.g., cooking in New York) may merit targeted follow-up using local data and inventory-component evaluation. Last, our results do not, by themselves, identify causal mechanisms or isolate the effects of specific regulatory interventions; rather, they provide a consistent baseline against which policies, interventions, and case studies can be evaluated.

If results here were used to estimate health impacts, that calculation would involve uncertainty in the concentration-response function (CRF). A prior study (*40*) found that among six well-established CRFs, variability is approximately a factor of 2 (see the Supplementary Materials). All six CFRs assume equal equitoxicity for all PM_2.5_. That attribute is uncertain; some research supports differential toxicity by source (*51*–*53*), much other research supports equal toxicity (*26*–*28*, *54*, *55*).

To quantitatively corroborate our findings, we compared our results against three literature-reported estimates of disparity by sector, year, and/or US geography: (i) Henneman *et al.* (*19*) reported PM_2.5_ from SO_2_ from coal electricity, 2002 to 2019, by US quadrant; (ii) Koolik *et al.* (*20*) reported total PM_2.5_ from on-road motor vehicle emissions in California (CA), 2002 to 2019; (iii) Liu *et al.* (*9*) reported disparity by state for total PM_2.5_, 2002 to 2010. The models used differ: Henneman: HyADS; Koolik: InMAP (with a CA-specific inventory, not EQUATES); Liu: empirical model.

The three comparisons reveal the following. First, absolute disparities in PM_2.5_ from coal-SO_2_ declined substantially during 2002 to 2019 [from 0.24 to 0.02 μg/m^3^ (our results); 0.20 to 0.01 μg/m^3^ (Henneman); see table S2]. In both studies (ours; Henneman), disparities are largest in the South. Considering disparities and their changes over time, by US quadrant, the correlation between Henneman’s and our results is 0.96. Second, for total PM_2.5_ from CA motor vehicles, absolute disparities declined during 2002 to 2019 [from 0.47 to 0.18 μg/m^3^ (our study); 0.35 to 0.18 μg/m^3^ (Koolik)], but relative disparities were essentially unchanged [~15% (ours); ~13% (Koolik)]. Third, considering changes in disparities for total PM_2.5_, during 2002 to 2010, the Spearman correlation between the Liu results and our results is 0.93 for national disparities and 0.73 for state-level disparities. All three prior articles report that absolute disparities declined while relative disparities were largely unchanged and also that disparities are greater by race-ethnicity than by income (i.e., consistent with findings #1 and #2). These consistencies with previous literature increase confidence in results presented here. Additional details are in the Supplementary Materials.

Our finding that exposure disparities are greater by race than by income is consistent with decades of research (*56*–*64*). To our knowledge, the earliest such documentation was in 1972, regarding pollution levels in 1960 in Kansas City, St. Louis, and Washington, DC (*65*–*67*). Our finding highlighting the importance of cooking emissions is consistent with source-resolved chemically speciated mobile monitoring in Oakland, CA, and Pittsburgh, PA, during 2016 to 2017 (*68*). That study concluded that in those two cities, exposure disparities are larger for cooking than for traffic emissions (*68*).

Results here aim to be useful to multiple audiences. Community groups and regulators can use the state-by-state evidence to prioritize which sectors merit closer investigation or targeted action. Researchers can use the dataset as a foundation for designing future case-study research, evaluating interventions, or probing underlying mechanisms of inequity. For example, Koolik *et al.* (*32*) show that absolute disparity (units: micrograms per cubic meter) can be expressed as the product of three components: sectoral emissions (grams per day); a population-average exposure factor that converts emissions into average exposure [(micrograms per cubic meter)/(grams per day)] and reflects atmospheric transport plus the average spatial relationship between sources and people; and a dimensionless relative disparity term that captures how unevenly exposures are distributed across groups within a state (*32*). Applying this decomposition to primary PM_2.5_ (see fig. S8 and the related Supplementary Materials) helps interpret drivers of change. For example, for motor vehicles, changes in absolute disparity are dominated by emission reductions. For other sectors (e.g., industrial and agriculture), other terms (relative disparity and exposure factor) play a larger role, consistent with factors such as changing source locations, population patterns, or within-state proximity between sources and affected communities. Together, these results imply that multiple possible approaches can reduce disparity, not only emission reduction.

Many causes (e.g., economic trends, population, technology developments, etc.) led to the environmental changes studied here; intentional changes will require action by local, state, and/or national governments. The Clean Air Act has driven transformative improvements in US air quality, largely by driving down industrial and transportation emissions (*29*, *30*, *40*, *69*–*71*); it was not designed to consider or address the disparities studied here (*33*, *34*). The prospects for national EJ policy may seem limited at present (2026), given the political leadership in the executive branch that is hostile to environmental improvement, EJ, and even to using in policymaking the sort of evidence presented here. However, any drive for improvements would be multidecadal, going beyond individual election cycles, and multilevel, reflecting that levels of engagement and action vary among states (*72*). Many of the current state-level EJ policies were enacted after 2019 (*72*), so their impacts are not observable here. Future research can investigate their effectiveness, such as by developing policy indices that can be measurable and uniform across states and time [e.g., (*72*–*74*)]. Policy contexts and opportunities across levels of government have changed and will continue to change; the sort of detailed work presented here can help guide policy.

## METHODS

Analyses in this study combine three main inputs (an air pollution model, a time-varying emission inventory, and Census data) using two main metrics (absolute disparity and relative disparity).

### Air pollution model

We use ISRM: the InMAP Source-Receptor Matrix (*31*). ISRM predicts annual-average PM_2.5_ concentrations across the contiguous United States attributable to anthropogenic emissions, resolved by five chemical species: primary PM_2.5_ and four species of secondary PM_2.5_ (particulate nitrate, particulate ammonium, particulate sulfate, and organic aerosol). ISRM predicts concentrations at a variably sized grid ranging from ~48 km in rural or remote areas to ~1 km in urban centers, with a nationwide population-weighted average grid resolution of ~7 km, sufficient to capture within-urban/equity-relevant exposure differences (*11*, *75*, *76*). The reported model performance is a mean fractional bias (MFB) of −38% and a mean fractional error (MFE) of 71% against observations; that comparison may reflect errors in modeling, the emission inventory, meteorology, and other inputs. Comparing against WRF-Chem, the population-weighted performance for InMAP is MFB = −6% and MFE = 18%; that comparison reflects InMAP’s ability to emulate WRF-Chem. All of those values meet the standard Boylan and Russell model performance criteria (“the level of accuracy that is considered to be acceptable for modeling applications”) for particulate matter (PM): MFB ⩽ ±60% and MFE ⩽ +75% (*77*). Additional model details, including extensive model-measurement comparisons, are provided by Tessum *et al.* (*47*). ISRM has been used extensively in the literature to understand PM_2.5_ concentrations and disparities (*78*–*84*); it is freely available online (*85*).

### Emission inventory

Emissions are from the US EPA EQUATES database (*86*), which harmonizes annual inventories for 2002 to 2019 into a consistent modeling framework. Traditional inventories are typically compiled separately for individual years; shifts in methodologies across versions can substantially complicate meaningful comparisons across years. In contrast, EQUATES applies consistent methods across time and space, thereby providing a coherent, nationwide dataset that enables robust analysis of long-term emission and exposure disparities. EQUATES is the most internally consistent long-term emission dataset of its type.

Uncertainties in emission inventories reflect limitations in, e.g., emission factors, activity data, and spatial surrogates. Studies suggest that uncertainties in emissions are on the order of tens of percent for SO_2_ and NO*_x_* and a factor of 2 or more for primary PM and black carbon (*87*–*91*). Agricultural and on-road NH_3_ emissions are often underestimated by 30% to a factor of 2 (*92*, *93*); historical mobile-source NO*_x_* and CO emissions calculated using average speed (e.g., EQUATES) are often overestimated relative to fuel-based methods (*94*, *95*). Spatial surrogates (e.g., population, land use, and road density) can introduce spatial errors such as shifts or misalignment. To maintain consistency with prior research and current best practices, our approach uses spatial surrogates from EPA and follows spatial allocation methods developed by EPA (*96*). We start from the same underlying National Emissions Inventory (NEI) emission data (e.g., county-level for area sources) that EPA’s Sparse Matrix Operator Kernel Emissions (SMOKE) model uses to produce EQUATES gridded emissions. We then apply the same EPA spatial surrogates and allocation methodology, implemented in Python, to grid emissions directly onto the ISRM variable-resolution source grid. Because our gridding follows the same process and surrogates as EQUATES, the resulting spatial allocation likely carries a comparable level of uncertainty as EQUATES. Exposure-based inferences here reflect the inherent uncertainties in emission estimates and their spatial allocation. Accordingly, these uncertainties are most likely to affect the magnitude and fine-scale spatial variability of sector-specific estimates but are less likely to alter the qualitative state-level patterns and the broad temporal trends. Uncertainties may differ across source sectors owing to inherent differences among sectors and their underlying data and methods.

EQUATES includes 5434 unique Source Classification Codes (SCCs) with nonzero emissions. We aggregated SCCs into 11 economic sectors, adapting the framework of Tessum *et al.* (*17*); categories contributing less than 1% to total PM_2.5_ direct or precursor emissions (e.g., other light-duty vehicles) were combined into broader sectors. The result is 11 sectors (percent contribution to total national year-2019 PM_2.5_ exposure): industrial (24%), agriculture (19%), residential combustion (10%), other sources (10%), gasoline vehicles (8%), cooking (5%), diesel vehicles (5%), offroad (5%), road dust (5%), electricity generation (5%), and construction (3%). “Cooking” refers to commercial cooking operations (e.g., restaurants) and includes emissions of primary PM_2.5_ (often organic aerosol) and associated pollutants from cooking activities and fuel use. “Agriculture” emissions include NH_3_ from fertilizer application and primary PM_2.5_ from agricultural dust; emissions from off-road agricultural equipment (e.g., tractors and harvesters: tailpipe emissions and soil/dust emissions) are classified as nonroad mobile sources.

### Census data

Demographic data are from the US Census Bureau for each year from 2002 to 2019 (*95*–*97*), combining the 2000 and 2010 Decennial Censuses with the American Community Survey (ACS) 5-year estimates through 2015 to 2019. Annual demographic estimates were generated to temporally match the emissions and concentration data for each analysis year by interpolating between available Census and ACS datasets. Race-ethnicity data were obtained from tract-level Census and ACS tables, providing population counts for non-Hispanic white, non-Hispanic Black, non-Hispanic Asian, Hispanic/Latino, and other/mixed groups (*97*, *98*). Household income data were obtained from ACS 5-year estimates (*99*) and categorized into tertiles for each state and year. For years before 2005, income data were interpolated between the 2000 Census and the earliest ACS 5-year release (2005 to 2009). We overlaid census tract polygons on top of ISRM grid cells and used the underlying ISRM grid to calculate population-weighted, group-specific PM_2.5_ exposures for each year. That is, our unit of analysis is census tract, with concentrations assigned on the basis of ISRM. For tracts overlapping multiple ISRM grid cells, we used an area-weighted average.

### Disparity metrics

Disparities are quantified for each state, sector, and year using two metrics. For each state, sector, and year, the reference population is the total population residing in that state (i.e., the population-weighted mean exposure across all Census-recorded residents in that state). (i) Absolute disparity is the difference in population-weighted average exposure (micrograms per cubic meter) between the most-exposed racial-ethnic group (or income group) and the overall population in that state. (ii) Relative disparity is the same difference but as a percentage (i.e. the absolute disparity divided by the population-weighted state average exposure).

By combining time-resolved emissions, state-level demographic data, and source-resolved air quality modeling, we assess how the contributions of each sector to racial-ethnic and income disparities in PM_2.5_ exposure have evolved over nearly two decades.

## Supplementary Materials

### This PDF file includes:

Figs. S1 to S8 Tables S1 and S2 References
