# Supplementary material for: PM2.5 air pollution inequities in the US by sector and state: Past trajectories and future directions
Source: Sci Adv. 2026 Jun 10;12(24):eaed7425. doi: 10.1126/sciadv.aed7425 (PMC13251869; doi:10.1126/sciadv.aed7425)
Supplement: Supplementary file 1 — Figs. S1 to S8 Tables S1 and S2 References [file sciadv.aed7425_sm.pdf]

Science Advances
AAAS

Supplementary Materials for

$PM_{2.5}$ air pollution inequities in the US by sector and state: Past trajectories and future directions

Bujin Bekbulat *et al.*

Corresponding author: Julian D. Marshall, jdmarsh@uw.edu

Sci. Adv. **12**, eaed7425 (2026)
DOI: 10.1126/sciadv.aed7425

This PDF file includes:

Figs. S1 to S8
Tables S1 and S2
References

The Supplement provides additional figures, tables, and text that extend the analyses in the main text.

Figures S1–S6 present results for racial-ethnic and income groups not shown in Figures 1–4. These results underscore that exposure disparities by income are generally smaller than those by race-ethnicity. Figures S5–S6 visualize contributions to exposure using variably sized tiles: Figure S5 highlights which sectors contribute most to exposures for racial-ethnic groups, Figure S6 is analogous for income groups. Figure S7 maps the most-exposed racial/ethnic group by state for each sector, showing spatial variation and shifts over time. Figure S8 summarizes percent changes in emissions, population-weighted exposure factors, and absolute and relative disparities, identifying which of these factors drive changes in absolute disparity for each sector.

Table S1 reports changes in absolute and relative disparities specifically for industrial-sector emissions, with full data for other sectors and states provided in the raw data files. Table S2 compares our results to Henneman et al. (19), confirming consistency in regional patterns while showing that racial-ethnic disparities remain larger than income-based disparities. Together, these figures and tables highlight the persistence, drivers, and geographic patterns of exposure disparities across demographic groups.

We next provide details regarding the sentence in the main text, "A prior study [41] found that among six well established CRFs, variability is approximately a factor of 2 (see SI)." That study [41] estimated $PM_{2.5}$ mortality using EQUATES, ISRM, and six CRFs [3,4, 26-28, 99]. Across the six central-tendency CRFs, mortality estimates varied from 52% lower (Wu et al. [26]) to 113% higher (Lepeule et al. [27]) than the core estimate (Orellano et al. [28]). Those results indicate that uncertainty across the six CRFs is approximately a factor of two. In comparison, for individual CRFs, 95% confidence intervals (CI) varied from +/- 12% (Wu et al. [26]) to +53/-48% (Lepeule et al. [28]).

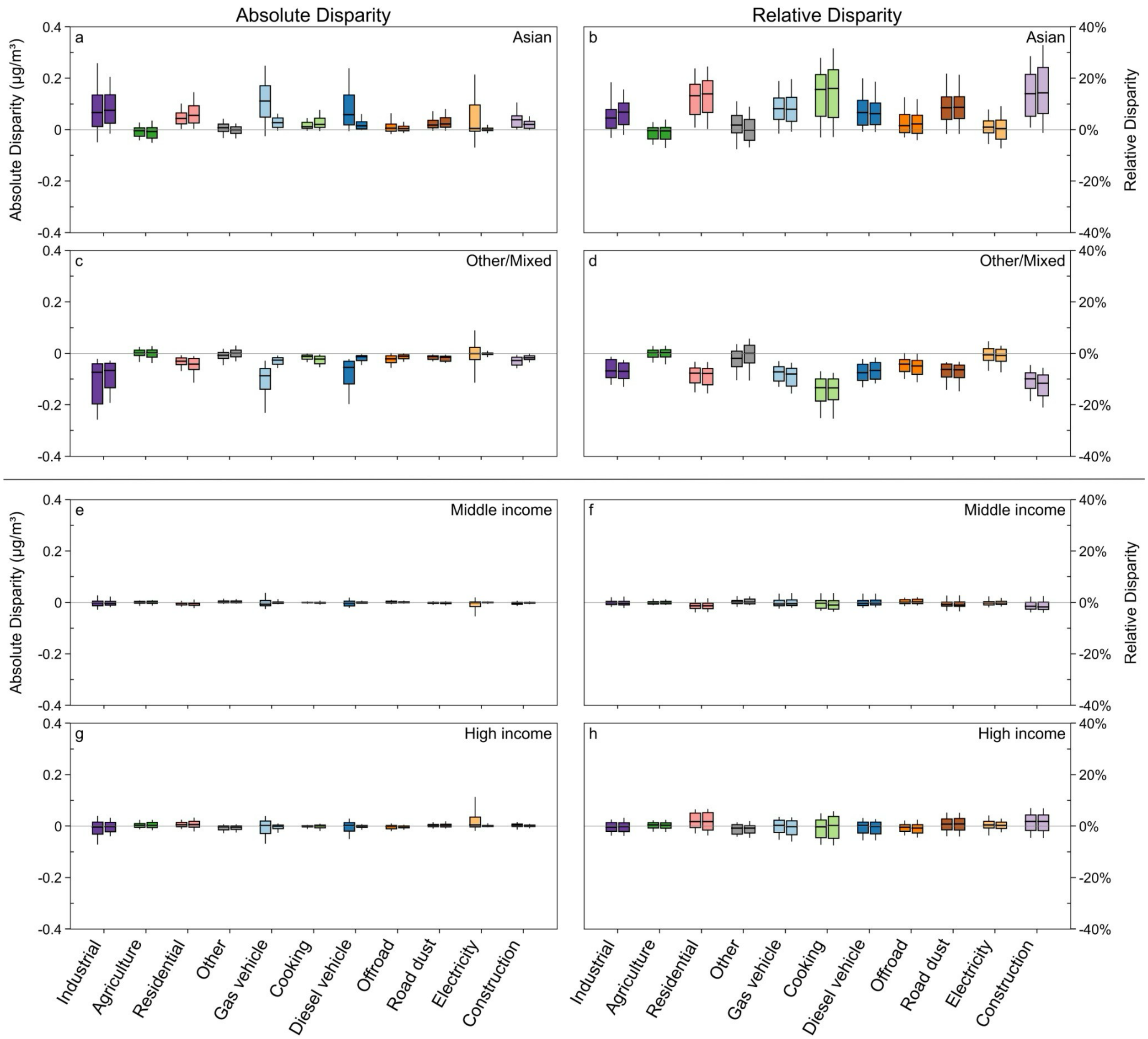

Figure S1. Results and display analogous to Figure 1, for additional racial-ethnic and income groups not included in Figure 1.

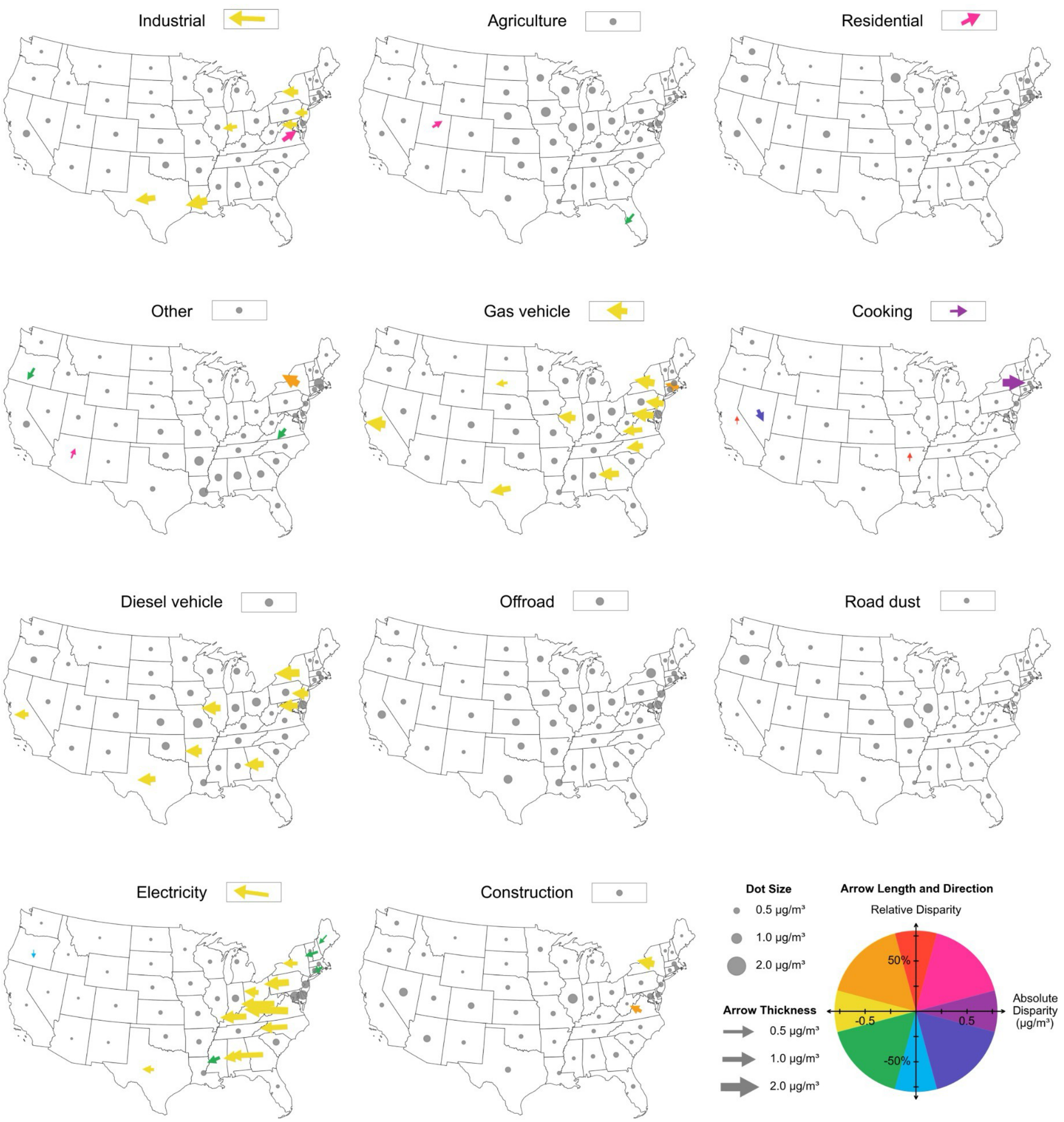

Figure S2. Results and display analogous to Figure 2, for income.

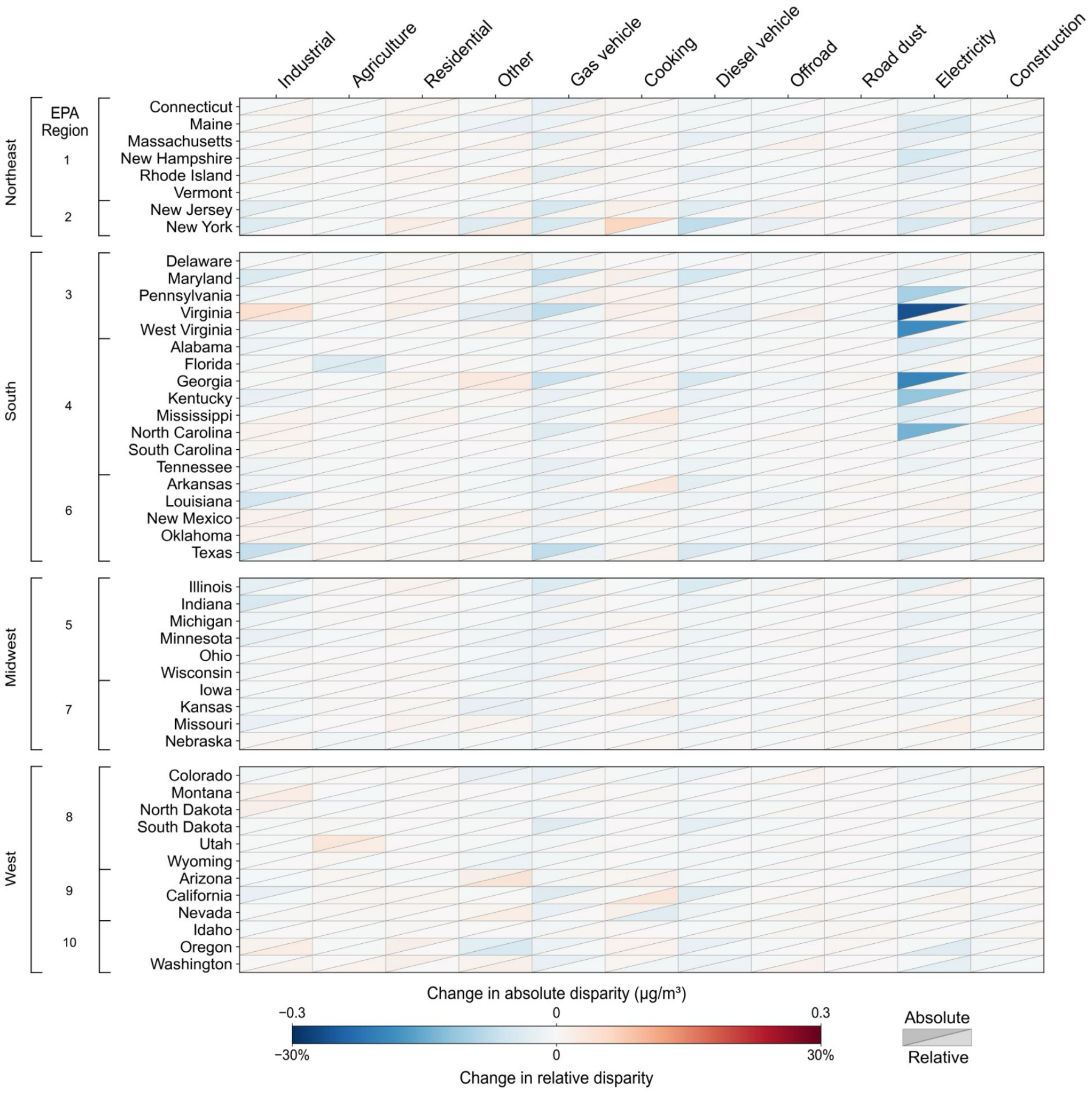

Figure S3. Results and display analogous to Figure 3, for income.

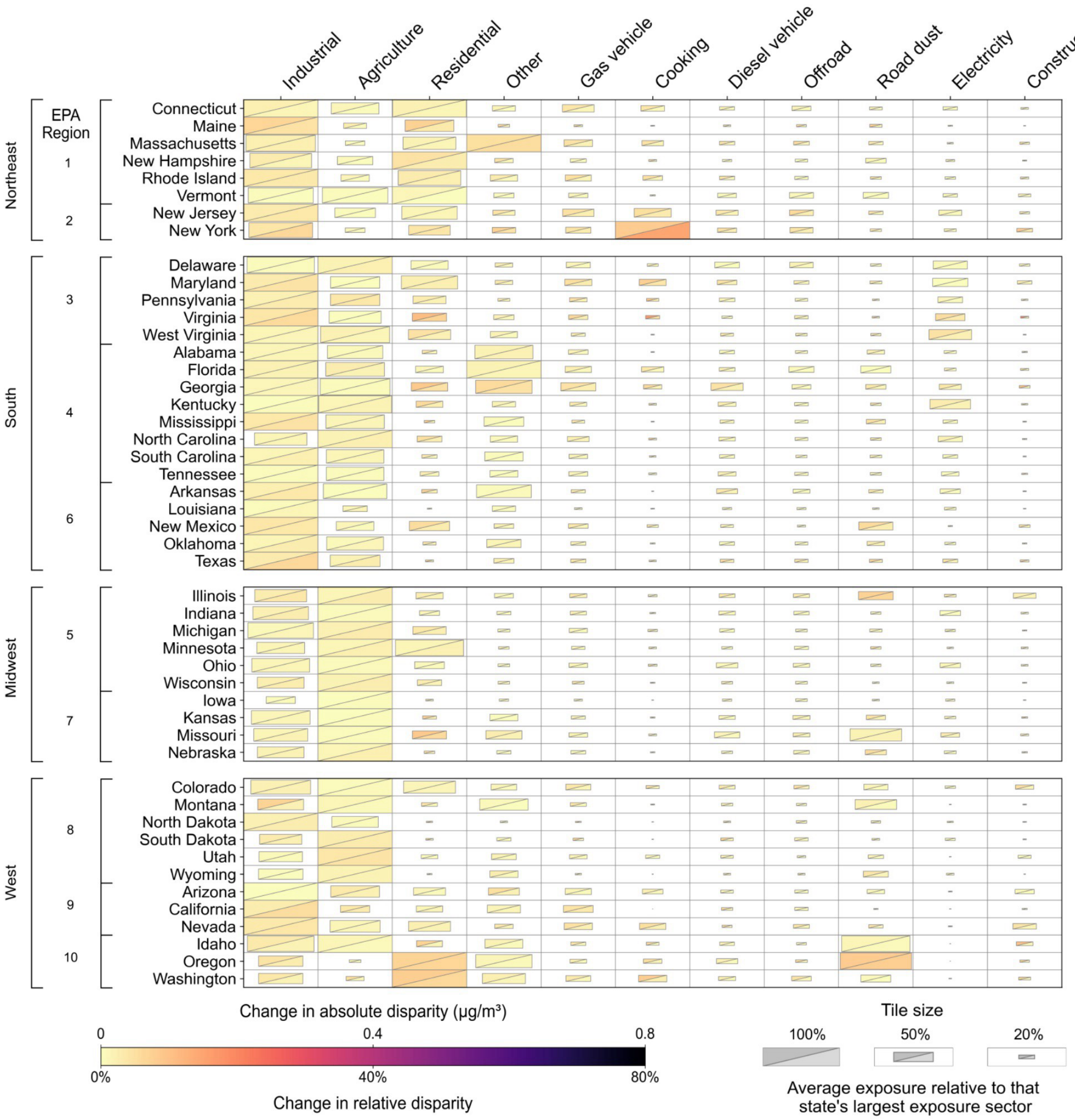

Figure S4. Results and display analogous to Figure 4, for income.

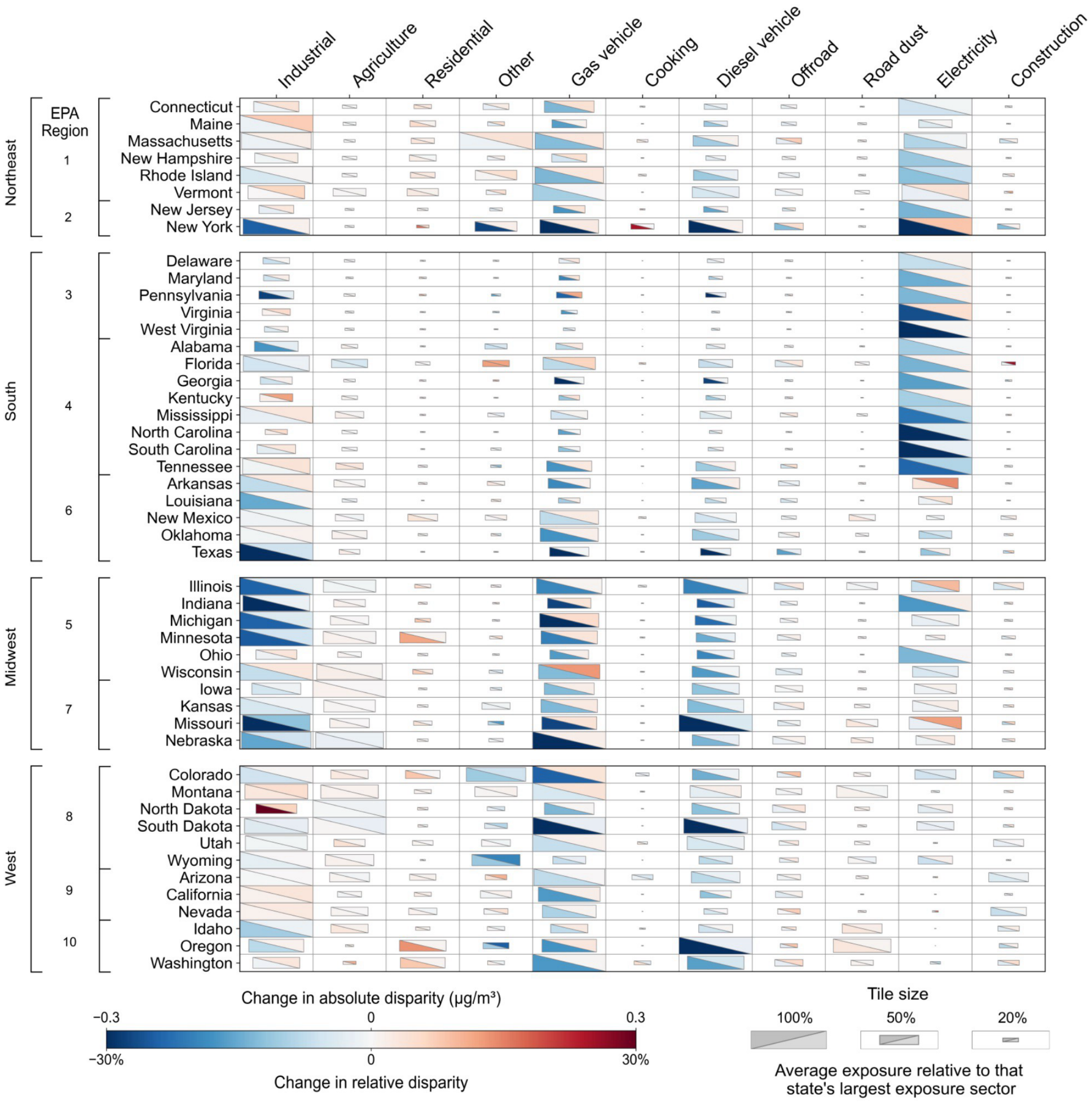

Figure S5. Results and display analogous to Figure 3, but with tile size proportional to exposure relative to maximum-exposure sector, i.e., this figure displays the data from Figure 3 using the display layout (variably-sized tiles) from Figure 4.

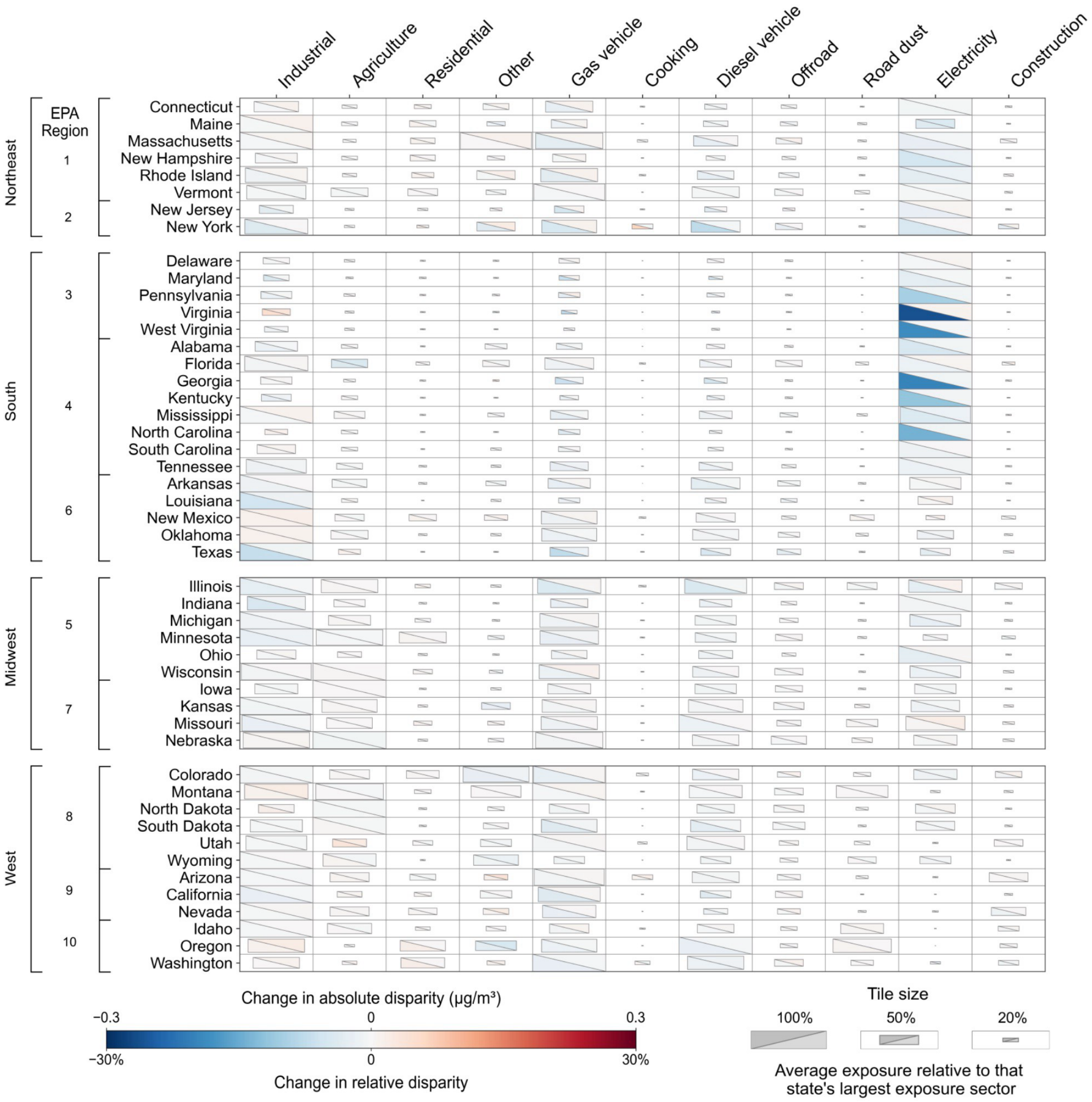

Figure S6. Results and display analogous to Figure S5, but for income.

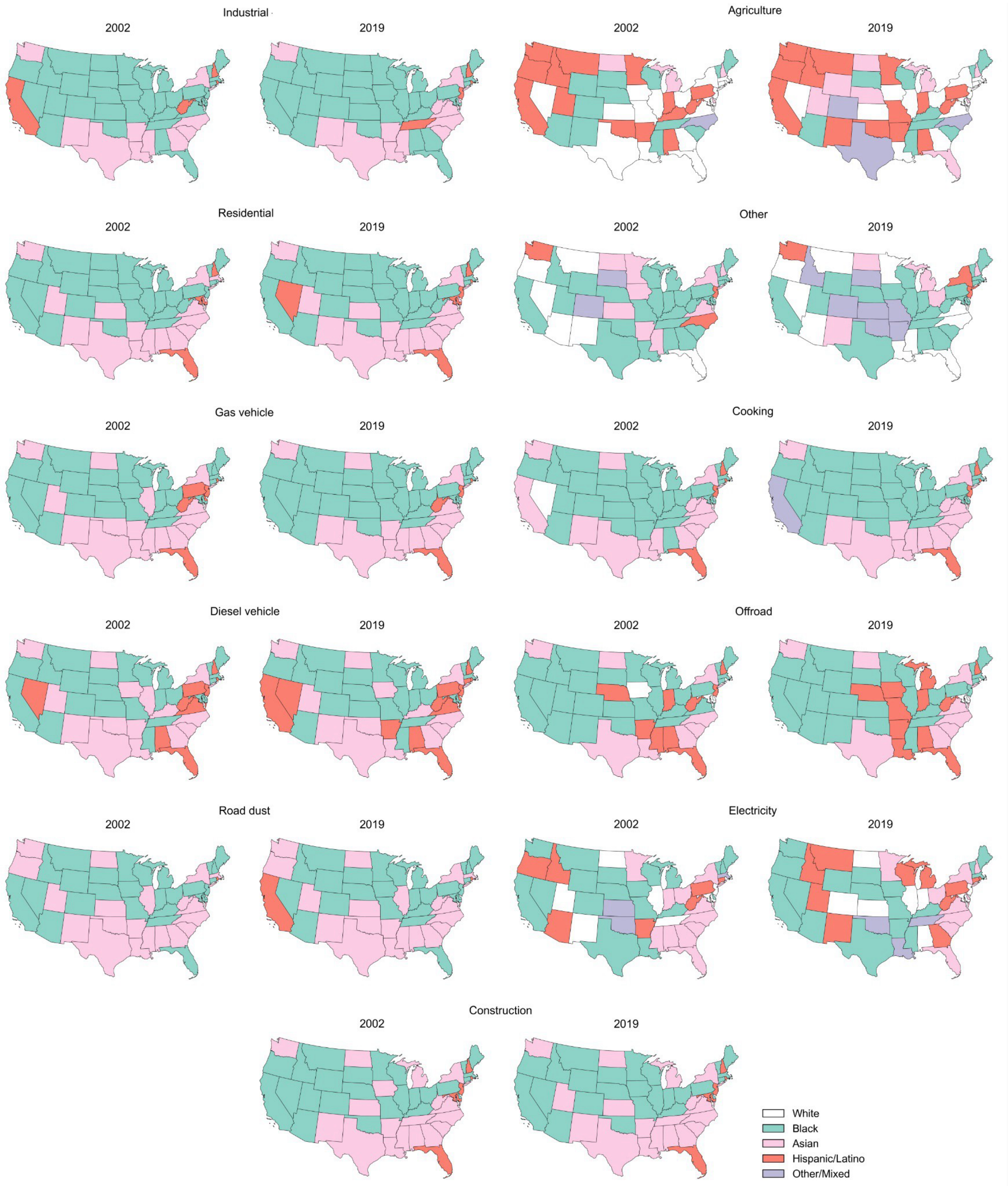

Figure S7. Most exposed racial/ethnic group by U.S. state for each emissions sector in 2002 (left) and 2019 (right). Each state is shaded by the racial/ethnic group with the highest exposure to $PM_{2.5}$ from that sector. Colors indicate the dominant exposed group (White, Black, Asian, Hispanic/Latino, Other/Mixed). The side-by-side layout highlights how disparities in exposure have shifted over time across sectors.

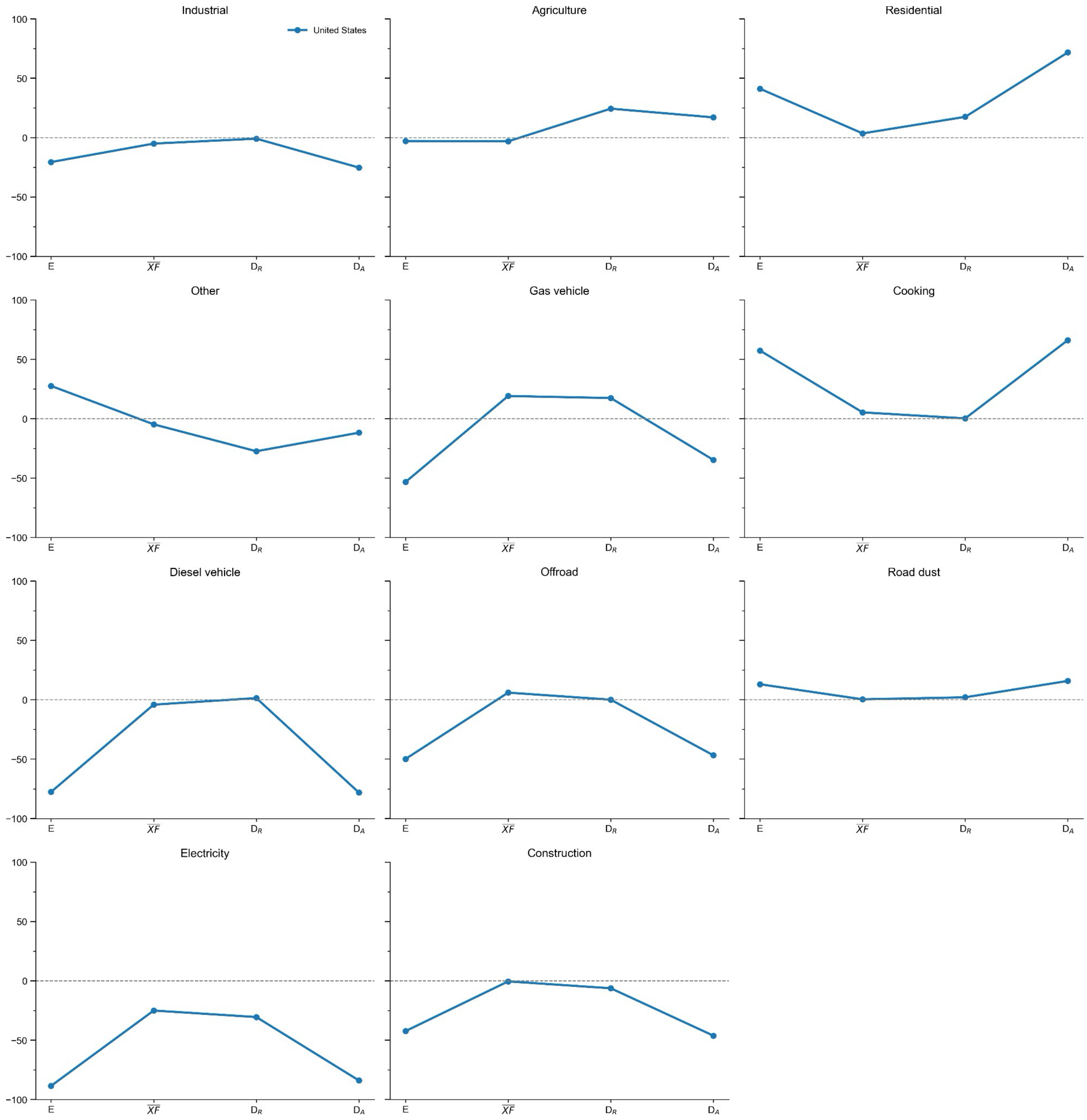

Figure S8. Percent changes in emissions and exposure disparity metrics by sector (2002–2019). Each panel shows the percent change in four metrics for primary $PM_{2.5}$: emissions (E), exposure factor (XF, i.e., population-weighted` concentration, divided by emissions), relative disparity (D_R), and absolute disparity (D_A). The blue line indicates the national (United States) population-weighted average. Positive values indicate increases and negative values indicate decreases between 2002 and 2019.

Table S1. Changes in absolute and relative exposure disparities for each racial/ethnic and income group, for industrial-sector emissions*, for the median U.S. state

Group	Absolute (μg/m^3)		Relative (%)	
	2002	2019	2002	2019
White	-0.18	-0.15	-12.37	-14.38
Black	0.14	0.11	10.79	11.78
Latino/Hispanic	0.04	0.03	2.41	3.27
Asian	0.07	0.07	4.49	6.80
Other	-0.08	-0.07	-6.84	-6.97
Low Income	0.0040	0.0036	0.53	0.48
Middle Income	-0.0020	-0.0028	-0.08	-0.30
High Income	-0.0020	-0.0010	-0.44	-0.19

* For other sectors and states, see data files.

Table S2. Comparison of changes in $PM_{2.5}$ exposure disparity* by race/ethnicity (White, Black, Asian, Hispanic, Native American, and Pacific Islander) across four U.S. regions

	Region	Disparity in 2002 Henneman et al. (2023) [8]	Disparity in 2019 Henneman et al. (2023) [8]	Disparity in 2002 (our results)	Disparity in 2019 (our results)
Absolute Disparity ($\mu g/m^3$)	Northeast	0.02	0.00	0.09	0.01
	South	0.20	0.01	0.24	0.02
	North Central	0.06	0.00	0.07	0.00
	West	0.01	0.00	0.02	0.00
Relative Disparity (%)	Northeast	10%	10%	9%	6%
	South	30%	27%	10%	10%
	North Central	8%	8%	9%	10%
	West	160%	160%	29%	30%

* For comparability ("apples-to-apples"), the analysis includes only populations with the same race/ethnicity categories and considers emissions exclusively from SO_2 originating in coal-fired power plants. Therefore, the results here differ from those presented in the main text, which include all emission sectors, 5 pollutants, and 5 racial ethnicity groups. Absolute disparity represents the difference in $PM_{2.5}$ exposure between the most exposed racial/ethnic group and the population average.

REFERENCES

1. United States Environmental Protection Agency, Benefits and Costs of the Clean Air Act 1990–2020, the Second Prospective Study (United States Environmental Protection Agency, 2015); www.epa.gov/clean-air-act-overview/benefits-and-costs-clean-air-act-1990-2020-second-prospective-study.

2. Global Burden of Disease, GBD Compare VizHub (Institute for Health Metrics and Evaluation, 2025); https://vizhub.healthdata.org/gbd-compare/.

3. C. A. Pope III, J. S. Lefler, M. Ezzati, J. D. Higbee, J. D. Marshall, S.-Y. Kim, M. Bechle, K. S. Gilliat, S. E. Vernon, A. L. Robinson, R. T. Burnett, Mortality risk and fine particulate air pollution in a large, representative cohort of U.S. adults. *Environ. Health Perspect.* **128**, 77007 (2020).

4. R. Burnett, H. Chen, M. Szyszkowicz, N. Fann, B. Hubbell, C. A. Pope III, J. S. Apte, M. Brauer, A. Cohen, S. Weichenthal, J. Coggins, Q. di, B. Brunekreef, J. Frostad, S. S. Lim, H. Kan, K. D. Walker, G. D. Thurston, R. B. Hayes, C. C. Lim, M. C. Turner, M. Jerrett, D. Krewski, S. M. Gapstur, W. R. Diver, B. Ostro, D. Goldberg, D. L. Crouse, R. V. Martin, P. Peters, L. Pinault, M. Tjepkema, A. van Donkelaar, P. J. Villeneuve, A. B. Miller, P. Yin, M. Zhou, L. Wang, N. A. H. Janssen, M. Marra, R. W. Atkinson, H. Tsang, T. Quoc Thach, J. B. Cannon, R. T. Allen, J. E. Hart, F. Laden, G. Cesaroni, F. Forastiere, G. Weinmayr, A. Jaensch, G. Nagel, H. Concin, J. V. Spadaro, Global estimates of mortality associated with long-term exposure to outdoor fine particulate matter. *Proc. Natl. Acad. Sci. U.S.A.* **115**, 9592–9597 (2018).

5. Q. Di, Y. Wang, A. Zanobetti, Y. Wang, P. Koutrakis, C. Choirat, F. Dominici, J. D. Schwartz, Air pollution and mortality in the Medicare population. *N. Engl. J. Med.* **376**, 2513–2522 (2017).

6. M. C. Turner, Z. J. Andersen, A. Baccarelli, W. R. Diver, S. M. Gapstur, C. A. Pope III, D. Prada, J. Samet, G. Thurston, A. Cohen, Outdoor air pollution and cancer: An overview of the current evidence and public health recommendations. *CA Cancer J. Clin.* **70**, 460–479 (2020).

7. I. Mikati, A. F. Benson, T. J. Luben, J. D. Sacks, J. Richmond-Bryant, Disparities in distribution of particulate matter emission sources by race and poverty status. *Am. J. Public Health* **108**, 480–485 (2018).

8. A. Jbaily, X. Zhou, J. Liu, T. H. Lee, L. Kamareddine, S. Verguet, F. Dominici, J. D. Schwartz, Air pollution exposure disparities across U.S. population and income groups. *Nature* **601**, 228–233 (2022).

9. J. Liu, L. P. Clark, M. J. Bechle, A. Hajat, S. Y. Kim, A. L. Robinson, L. Sheppard, A. A. Szpiro, J. D. Marshall, Disparities in air pollution exposure in the United States by race/ethnicity and income 1990–2010. *Environ. Health Perspect.* **129**, 127005 (2021).

10. T. W. Collins, S. E. Grineski, Y. Shaker, C. J. Mullen, Communities of color are disproportionately exposed to long-term and short-term $PM_{2.5}$ in metropolitan America. *Environ. Res.* **214**, 114038 (2022).

11. D. A. Paolella, C. W. Tessum, P. J. Adams, J. S. Apte, S. Chambliss, J. Hill, N. Z. Mueller, J. D. Marshall, Effect of model spatial resolution on estimates of fine particulate matter exposure and exposure disparities in the United States. *Environ. Sci. Technol. Lett.* **5**, 436–441 (2018).

12. J. D. Marshall, A. Giang, C. Ivey, Tracing environmental equity and air pollution research at ES&T. *Environ. Sci. Technol.* **60**, 5905–5909 (2026).

13. J. D. Marshall, L. H. Koolik, A. Unal, R. Morello-Frosch, J. S. Apte, Advancing methods and models that promote equity in ambient air quality. *Annu. Rev. Public Health* **47**, 283–303 (2026).

14. J. Colmer, I. Hardman, J. Shimshack, J. Voorheis, Disparities in $PM_{2.5}$ air pollution in the United States. *Science* **369**, 575–578 (2020).

15. United States Environmental Protection Agency, EPA's environmental justice strategic plan: 2022–2026 (2022); https://www.epa.gov/system/files/documents/2022-03/fy-2022-2026-epa-strategic-plan.pdf [accessed 2 November 2024].

16. California Office of Environmental Health Hazard Assessment, CalEnviroScreen 4.0 (2021); https://oehha.ca.gov/calenviroscreen/report/calenviroscreen-40.

17. C. W. Tessum, J. S. Apte, A. L. Goodkind, N. Z. Muller, K. A. Mullins, D. A. Paolella, S. Polasky, N. P. Springer, S. V. Thakrar, J. D. Hill, Inequity in consumption of goods and services adds to racial–ethnic disparities in air pollution exposure. *Proc. Natl. Acad. Sci. U.S.A.* **116**, 6001–6006 (2019).

18. C. W. Tessum, D. A. Paolella, S. E. Chambliss, J. S. Apte, J. D. Hill, J. D. Marshall, $PM_{2.5}$ polluters disproportionately and systemically affect people of color in the United States. *Sci. Adv.* **7**, eabf4491 (2021).

19. L. R. F. Henneman, M. M. Rasel, C. Choirat, S. C. Anenberg, C. Zigler, Inequitable exposures to U.S. coal power plant–related $PM_{2.5}$: 22 years and counting. *Environ. Health Perspect.* **131**, 037005 (2023).

20. L. H. Koolik, Á. Alvarado, A. Budahn, L. Plummer, J. D. Marshall, J. S. Apte, $PM_{2.5}$ exposure disparities persist despite strict vehicle emissions controls in California. *Sci. Adv.* **10**, eadn8544 (2024).

21. Y. Nunez, J. Benavides, J. A. Shearston, E. M. Krieger, M. Daouda, L. R. F. Henneman, E. E. McDuffie, J. Goldsmith, J. A. Casey, M. A. Kioumourtzoglou, An environmental justice analysis of air pollution emissions in the United States from 1970 to 2010. *Nat. Commun.* **15**, 268 (2024).

22. T. Goforth, D. Nock, Air pollution disparities and equality assessments of U.S. national decarbonization strategies. *Nat. Commun.* **13**, 7488 (2022).

23. K. H. Jordan, L. R. Dennin, P. J. Adams, P. Jaramillo, N. Z. Muller, Climate policy reduces racial disparities in air pollution from transportation and power generation. *Environ. Sci. Technol.* **58**, 21510–21522 (2024).

24. P. Picciano, M. Qiu, S. D. Eastham, M. Yuan, J. Reilly, N. E. Selin, Air quality related equity implications of U.S. decarbonization policy. *Nat. Commun.* **14**, 5543 (2023).

25. P. Polonik, K. Ricke, S. Reese, J. Burney, Air quality equity in U.S. climate policy. *Proc. Natl. Acad. Sci. U.S.A.* **120**, e2217124120 (2023).

26. X. Wu, D. Braun, J. Schwartz, M. A. Kioumourtzoglou, F. Dominici, Evaluating the impact of long-term exposure to fine particulate matter on mortality among the elderly. *Sci. Adv.* **6**, eaba5692 (2020).

27. J. Lepeule, F. Laden, D. Dockery, J. Schwartz, Chronic exposure to fine particles and mortality: An extended follow-up of the Harvard Six Cities study from 1974 to 2009. *Environ. Health Perspect.* **120**, 965–970 (2012).

28. P. Orellano, E. Samoli, R. Pérez Velasco, M. I. Kasdagli, Long-term exposure to particulate matter and mortality: An update of the WHO air quality guidelines systematic review and meta-analysis. *Int. J. Public Health* **69**, 1607683 (2024).

29. S. Rosanka, A. M. Carlton, The legacy of the U.S. Clean Air Act at a crossroads. *npj Clean Air* **1**, 16 (2025).

30. G. D. Thurston, R. C. Gwynn, M. Frampton, History and future outlook for the U.S. Clean Air Act. *Ann. Am. Thorac. Soc.* **23**, 34–37 (2026).

31. L. Koolik, S. Speizer, C. Rong, S. Chambliss, J. Marshall, R. Morello-Frosch, C. Tessum, J. Apte, Methodological design choices can affect air pollution exposure disparity estimates: A case study on California's agricultural sector. *Environ. Sci. Technol.* **60**, 4753–4763 (2026).

32. L. H. Koolik, R. D. Bullard, E. Min, R. Morello-Frosch, R. Patterson, M. Salgado, N. Wedekind, J. D. Marshall, J. S. Apte, Eliminating air pollution disparities requires more than emission reduction. *Proc. Natl. Acad. Sci. U.S.A.* **122**, e2505888122 (2025).

33. Y. Wang, J. S. Apte, J. D. Hill, C. E. Ivey, D. Johnson, E. Min, R. Morello-Frosch, R. Patterson, A. L. Robinson, C. W. Tessum, J. D. Marshall, Air quality policy should quantify effects on disparities. *Science* **381**, 272–274 (2023).

34. Y. Wang, J. S. Apte, J. D. Hill, C. E. Ivey, R. F. Patterson, A. L. Robinson, C. W. Tessum, J. D. Marshall, Location-specific strategies for eliminating US national racial-ethnic $PM_{2.5}$ exposure inequality. *Proc. Natl. Acad. Sci. U.S.A.* **119**, e2205548119 (2022).

35. M. P. S. Thind, C. W. Tessum, I. L. Azevedo, J. D. Marshall, Fine particulate air pollution from electricity generation in the US: Health impacts by race, income, and geography. *Environ. Sci. Technol.* **53**, 14010–14019 (2019).

36. M. P. S. Thind, C. W. Tessum, J. D. Marshall, Environmental health, racial/ethnic health disparity, and climate impacts of inter-regional freight transport in the United States. *Environ. Sci. Technol.* **57**, 884–895 (2023).

37. E. G. Dimanchev, S. Paltsev, M. Yuan, D. Rothenberg, C. W. Tessum, J. D. Marshall, N. E. Selin, Health co-benefits of sub-national renewable energy policy in the US. *Environ. Res. Lett.* **14**, 085012 (2019).

38. T. Fried, C. Tejada, S. Dennis-Bauer, O. Boldbaatar, A. Goodchild, J. D. Marshall, L. García, Logistics of zoning, zoning for logistics: Toward healthy and equitable development for urban freight. *J. Am. Plann. Assoc.* **92**, 1–18 (2026).

39. L. Liu, T. Hwang, S. Lee, Y. Ouyang, B. Lee, S. J. Smith, C. W. Tessum, J. D. Marshall, F. Yan, K. Daenzer, T. C. Bond, Health and climate impacts of future United States land freight modelled with global-to-urban models. *Nat. Sustain.* **2**, 105–112 (2019).

40. B. Bekbulat, A. Unal, A. Sharma, J. S. Apte, J. D. Marshall, Changes in $PM_{2.5}$-attributable mortality in the United States by sector, 2002–2019. *Environ. Sci. Technol. Lett.* **12**, 1644–1650 (2025).

41. A. L. Goodkind, C. W. Tessum, J. Coggins, J. D. Hill, J. D. Marshall, Fine-scale damage estimates of particulate matter air pollution reveal opportunities for location-specific mitigation of emissions. *Proc. Natl. Acad. Sci. U.S.A.* **116**, 8775–8780 (2019).

42. S. K. Thakrar, S. Balasubramanian, P. J. Adams, I. M. L. Azevedo, N. Z. Muller, S. N. Pandis, S. Polasky, C. A. Pope III, A. L. Robinson, J. S. Apte, C. W. Tessum, J. D. Marshall,

J. D. Hill, Reducing mortality from air pollution in the United States by targeting specific emission sources. *Environ. Sci. Technol. Lett.* **7**, 639–645 (2020).

43. S. Balasubramanian, N. G. G. Domingo, N. D. Hunt, M. Gittlin, K. K. Colgan, J. D. Marshall, A. L. Robinson, I. M. L. Azevedo, S. K. Thakrar, M. A. Clark, C. W. Tessum, P. J. Adams, S. N. Pandis, J. D. Hill, The food we eat, the air we breathe: A review of the fine particulate matter-induced air quality health impacts of the global food system. *Environ. Res. Lett.* **16**, 103004 (2021).

44. N. G. G. Domingo, S. Balasubramanian, S. K. Thakrar, M. A. Clark, P. J. Adams, J. D. Marshall, N. Z. Muller, S. N. Pandis, S. Polasky, A. L. Robinson, C. W. Tessum, D. Tilman, P. Tschofen, J. D. Hill, Air quality–related health damages of food. *Proc. Natl. Acad. Sci. U.S.A.* **118**, e2013637118 (2021).

45. K. K. Shlipak, S. F. Camilleri, V. A. Lang, A. Montgomery, J. L. Schnell, D. E. Horton, Ambient air quality and health impacts of $PM_{2.5}$ from US residential wood combustion. *Sci. Adv.* **12**, eadz0189 (2026).

46. F. J. Ries, J. D. Marshall, M. Brauer, Intake fraction of urban wood smoke. *Environ. Sci. Technol.* **43**, 4701–4706 (2009).

47. C. W. Tessum, J. D. Hill, J. D. Marshall, InMAP: A model for air pollution interventions. *PLOS ONE* **12**, e0176131 (2017).

48. E. A. Gilmore, J. Heo, N. Z. Muller, C. W. Tessum, J. D. Hill, J. D. Marshall, P. J. Adams, An inter-comparison of air quality social cost estimates from reduced-complexity models. *Environ. Res. Lett.* **14**, 074016 (2019).

49. C. L. Gallagher, T. Holloway, C. W. Tessum, C. M. Jackson, C. Heck, Combining satellite-derived $PM_{2.5}$ data and a reduced-form air quality model to support air quality analysis in US cities. *GeoHealth* **7**, e2023GH000788 (2023).

50. H. Özkaynak, G. D. Thurston, Associations between 1980 U.S. mortality rates and alternative measures of airborne particle concentration. *Risk Anal.* **7**, 449–461 (1987).

51. M. A. Kioumourtzoglou, B. A. Coull, F. Dominici, P. Koutrakis, J. Schwartz, H. Suh, The impact of source contribution uncertainty on the effects of source-specific $PM_{2.5}$ on hospital admissions: A case study in Boston, MA. *J. Expo. Sci. Environ. Epidemiol.* **24**, 365–371 (2014).

52. H. Zheng, D. Wu, S. Wang, X. Li, L. N. Jin, B. Zhao, S. Li, Y. Sun, Z. Dong, Q. Wu, X. Chen, Y. Liu, J. Chen, H. Tian, Q. Liu, J. Jiang, H. Kan, K. He, H. He, C. Chen, J. Zhao, S. Weichenthal, J. S. Ji, A. J. Cohen, J. Hao, Q. Li, Control of toxicity of fine particulate matter emissions in China. *Nature* **643**, 404–411 (2025).

53. P. H. Fischer, M. Marra, C. B. Ameling, G. J. M. Velders, R. Hoogerbrugge, W. de Vries, J. Wesseling, N. A. H. Janssen, D. Houthuijs, Particulate air pollution from different sources and mortality in 7.5 million adults — The Dutch Environmental Longitudinal Study (DUELS). *Sci. Total Environ.* **705**, 135778 (2020).

54. M. Miller, M. Clift, R. Harrison, F. Kelly, A. Doutsi, C. Mitsakou, N. Earl, K. Exley, A. Gowers, 57 particles in outdoor air: Are they all equal? *Ann. Work Expo. Health.* **67**, i81–i82 (2023).

55. L. P. Clark, D. B. Millet, J. D. Marshall, National patterns in environmental injustice and inequality: Outdoor NO_2 air pollution in the United States. *PLOS ONE* **9**, e94431 (2014).

56. R. D. Bullard, *Dumping in Dixie: Race, Class, and Environmental Quality* (Westview Press, 2000).

57. L. Downey, B. Hawkins, Race, income, and environmental inequality in the United States. *Sociol. Perspect.* **51**, 759–781 (2008).

58. P. Mohai, P. M. Lantz, J. Morenoff, J. S. House, R. P. Mero, Racial and socioeconomic disparities in residential proximity to polluting industrial facilities: Evidence from the Americans' Changing Lives Study. *Am. J. Public Health* **99**, S649–S656 (2009).

59. L. Downey, Environmental injustice: Is race or income a better predictor? *Soc. Sci. Q.* **79**, 766–778 (1998).

60. P. de Souza, S. Anenberg, C. Makarewicz, M. Shirgaokar, F. Duarte, C. Ratti, J. L. Durant, P. L. Kinney, D. Niemeier, Quantifying disparities in air pollution exposures across the United States using home and work addresses. *Environ. Sci. Technol.* **58**, 280–290 (2024).

61. Z. Li, D. M. Konisky, N. Zirogiannis, Racial, ethnic, and income disparities in air pollution: A study of excess emissions in Texas. *PLOS ONE* **14**, e0220696 (2019).

62. S. Beard, K. Freeman, M. L. Velasco, W. Boyd, T. Chamberlain, A. Latoni, D. Lasko, R. M. Lunn, L. O'Fallon, J. Packenham, M. M. Smarr, R. Arnette, C. Cavalier-Keck, J. Keck, N. Muhammad, O. Wilson, B. Wilson, A. Wilson, D. Dixon, Racism as a public health issue in environmental health disparities and environmental justice: Working toward solutions. *Environ. Health* **23**, 8 (2024).

63. M. J. Cheeseman, B. Ford, S. C. Anenberg, M. J. Cooper, E. V. Fischer, M. S. Hammer, S. Magzamen, R. V. Martin, A. van Donkelaar, J. Volckens, J. R. Pierce, Disparities in air pollutants across racial, ethnic, and poverty groups at US public schools. *GeoHealth* **6**, e2022GH000672 (2022).

64. M. D. Van Arsdol Jr., G. Sabagh, F. Alexander, Reality and the perception of environmental hazards. *J. Health Hum. Behav.* **5**, 144–153 (1964).

65. M. D. Van Arsdol Jr., Metropolitan growth and environmental hazards: An illustrative case (World Population Conference). *Ekistics* **21**, 48–50 (1966).

66. A. M. Freeman III, "The distribution of environmental quality," in *Environmental Quality Analysis: Theory and Method in the Social Sciences*, A. V. Kneese, B. T. Bower, Eds. (RFF Press, 1972), pp. 243–278.

67. R. U. Shah, E. S. Robinson, P. Gu, J. S. Apte, J. D. Marshall, A. L. Robinson, A. A. Presto, Socio-economic disparities in exposure to urban restaurant emissions are larger than for traffic. *Environ. Res. Lett.* **15**, 114039 (2020).

68. D. A. Ridley, C. L. Heald, K. J. Ridley, J. H. Kroll, Causes and consequences of decreasing atmospheric organic aerosol in the United States. *Proc. Natl. Acad. Sci. U.S.A.* **115**, 290–295 (2018).

69. J. L. Hand, A. J. Prenni, S. Copeland, B. A. Schichtel, W. C. Malm, Thirty years of the Clean Air Act Amendments: Impacts on haze in remote regions of the United States (1990–2018). *Atmos. Environ.* **243**, 117865 (2020).

70. R. Schmalensee, R. N. Stavins, Policy evolution under the Clean Air Act. *J. Econ. Perspect.* **33**, 27–50 (2019).

71. Environmental Justice State by State Law Library & Database, American University, Environmental Justice Clinic at Vermont Law School, Lone Star Legal Aid, Taproot Earth, Tishman Environment & Design Center at The New School, UC College of the Law, San Francisco, University of Texas School of Law, Austin Environmental Clinic, Environmental justice state by state: Law & policy database (2025); https://ejstatebystate.org/.

72. G. Samari, A. Nagle, K. Coleman-Minahan, Measuring structural xenophobia: US state immigration policy climates over ten years. *SSM Popul Health* **16**, 100938 (2021).

73. G. Zhang, Z. Chen, J. Li, B. Su, Y. Gao, L. Yu, Quantifying U.S. air pollution policy: How political and regional factors influence pollutant mitigation. *PNAS Nexus* **3**, pgae199 (2024).

74. S. E. Chambliss, C. P. R. Pinon, K. P. Messier, B. LaFranchi, C. R. Upperman, M. M. Lunden, A. L. Robinson, J. D. Marshall, J. S. Apte, Local- and regional-scale racial and ethnic disparities in air pollution determined by long-term mobile monitoring. *Proc. Natl. Acad. Sci. U.S.A.* **118**, e2109249118 (2021).

75. L. P. Clark, M. H. Harris, J. S. Apte, J. D. Marshall, National and intraurban air pollution exposure disparity estimates in the United States: Impact of data-aggregation spatial scale. *Environ. Sci. Technol. Lett.* **9**, 786–791 (2022).

76. J. W. Boylan, A. G. Russell, PM and light extinction model performance metrics, goals, and criteria for three-dimensional air quality models. *Atmos. Environ.* **40**, 4946–4959 (2006).

77. E. F. Choma, L. A. Robinson, K. C. Nadeau, Adopting electric school buses in the United States: Health and climate benefits. *Proc. Natl. Acad. Sci. U.S.A.* **121**, e2320338121 (2024).

78. E. F. Choma, J. S. Evans, J. A. Gómez-Ibáñez, Q. Di, J. D. Schwartz, J. K. Hammitt, J. D. Spengler, Health benefits of decreases in on-road transportation emissions in the United States from 2008 to 2017. *Proc. Natl. Acad. Sci. U.S.A.* **118**, e2107402118 (2021).

79. M. Qiu, C. M. Zigler, N. E. Selin, Impacts of wind power on air quality, premature mortality, and exposure disparities in the United States. *Sci. Adv.* **8**, eabn8762 (2022).

80. G. Guidi, F. Dominici, N. Steinsultz, G. Dance, L. Henneman, H. Richardson, E. Castro, F. J. Bargagli-Stoffi, S. Delaney, The environmental burden of the United States' bitcoin mining boom. *Nat. Commun.* **16**, 2970 (2025).

81. R. Deshmukh, P. Weber, O. Deschenes, D. Hernandez-Cortes, T. Kordell, R. Lee, C. Malloy, T. Mangin, M. Meng, S. Sum, V. Thivierge, A. Uppal, D. W. Lea, K. C. Meng, Equitable low-carbon transition pathways for California's oil extraction. *Nat. Energy* **8**, 597–609 (2023).

82. S. K. Thakrar, J. A. Johnson, S. Polasky, Land-use decisions have substantial air quality health effects. *Environ. Sci. Technol.* **58**, 381–390 (2024).

83. B. J. Sergi, P. J. Adams, N. Z. Muller, A. L. Robinson, S. J. Davis, J. D. Marshall, I. L. Azevedo, Optimizing emissions reductions from the U.S. power sector for climate and health benefits. *Environ. Sci. Technol.* **54**, 7513–7523 (2020).

84. A. L. Goodkind, C. W. Tessum, J. S. Coggins, J. D. Hill, J. D. Marshall, InMAP Source–Receptor Matrix (ISRM) dataset, version 1.2.1, Zenodo (2019); https://doi.org/10.5281/zenodo.2589760.

85. United States Environmental Protection Agency, EQUATES: A multi-year air quality modeling platform (2022); https://www.epa.gov/cmaq/equates.

86. Y. Zhao, C. P. Nielsen, Y. Lei, M. B. McElroy, J. Hao, Quantifying the uncertainties of a bottom-up emission inventory of anthropogenic atmospheric pollutants in China. *Atmos. Chem. Phys.* **11**, 2295–2308 (2011).

87. T. C. Bond, D. G. Streets, K. F. Yarber, S. M. Nelson, J. H. Woo, Z. Klimont, A technology-based global inventory of black and organic carbon emissions from combustion. *J. Geophys. Res. Atmos.* **109**, D14203 (2004).

88. Y. Ren, C. R. Oxford, D. Zhang, X. Liu, H. Zhu, A. M. Dillner, W. H. White, R. K. Chakrabarty, S. Hasheminassab, D. J. Diner, E. J. le Roy, J. Kumar, V. Viteri, K. Song, C. Akoshile, O. Amador-Muñoz, A. Asfaw, R. Y. W. Chang, D. Francis, P. Gahungu, R. M. Garland, M. Grutter, J. Kim, K. Langerman, P. C. Lee, P. Lestari, O. L. Mayol-Bracero, M. Naidoo, N. Nelli, N. O'Neill, S. S. Park, A. Salam, B. Sarangi, Y. Schechner, R. Schofield, S. N. Tripathi, E. Windwer, M. T. Wu, Q. Zhang, Y. Rudich, M. Brauer, R. V. Martin, Black carbon emissions generally underestimated in the Global South as revealed by globally distributed measurements. *Nat. Commun.* **16**, 7010 (2025).

89. S. Wang, L. Guan, J. Cohen, K. Qin, Reconstructing top-down global black carbon emissions using remote sensing and models. *Atmos. Pollut. Res.* **16**, 102633 (2025).

90. T. Rönkkö, S. Saarikoski, N. Kuittinen, P. Karjalainen, H. Keskinen, A. Järvinen, F. Mylläri, P. Aakko-Saksa, H. Timonen, Review of black carbon emission factors from different anthropogenic sources. *Environ. Res. Lett.* **18**, 033004 (2023).

91. H. Cao, D. Henze, M. Shephard, E. Dammers, K. Cady-Pereira, M. Alvarado, C. Lonsdale, G. Luo, F. Yu, L. Zhu, C. Danielson, E. Edgerton, Inverse modeling of NH_3 sources using CrIS remote-sensing measurements. *Environ. Res. Lett.* **15**, 104046 (2020).

92. N. J. Farren, J. Davison, R. A. Rose, R. L. Wagner, D. C. Carslaw, Underestimated ammonia emissions from road vehicles. *Environ. Sci. Technol.* **54**, 15689–15697 (2020).

93. B. C. McDonald, S. A. McKeen, Y. Y. Cui, R. Ahmadov, S.-W. Kim, G. J. Frost, I. B. Pollack, J. Peischl, T. B. Ryerson, J. S. Holloway, M. Graus, C. Warneke, J. B. Gilman, J. A. de Gouw, J. Kaiser, F. N. Keutsch, T. F. Hanisco, G. M. Wolfe, M. Trainer, Modeling ozone in the eastern U.S. using a fuel-based mobile source emissions inventory. *Environ. Sci. Technol.* **52**, 7360–7370 (2018).

94. C. Harkins, B. C. McDonald, D. K. Henze, C. Wiedinmyer, A fuel-based method for updating mobile-source emissions during the COVID-19 pandemic. *Environ. Res. Lett.* **16**, 054029 (2021).

95. CMAS Center, "CMAQ"; https://www.cmascenter.org/cmaq/index.cfm.

96. United States Census Bureau, Summary file 1 dataset (United States Department of Commerce, Washington, DC, 2001); https://www.census.gov/data/datasets/2000/dec/summary-file-1.html.

97. United States Census Bureau, Summary file 1 dataset (United States Department of Commerce, Washington, DC, 2011); https://www.census.gov/data/datasets/2010/dec/summary-file-1.html.

98. United States Census Bureau, American Community Survey (ACS) (United States Department of Commerce, Washington, DC, 2005–2019); https://www.census.gov/programs-surveys/acs.

99. D. Krewski, M. Jerrett, R. T. Burnett, R. Ma, E. Hughes, Y. Shi, M. C. Turner, C. A. Pope III, G. Thurston, E. E. Calle, M. J. Thun, Extended follow-up and spatial analysis of the American Cancer Society study linking particulate air pollution and mortality. *Res. Rep. Health Eff. Inst.* **140**, 5–155 (2009).
